# The High Pressure Bogey

**Published:** 1914-05

**Authors:** 


**The High Pressure Bogey.** Solomon Solis Cohen, Med. Rev. of Revs., Feb., 1914, contends that the sphygomanometer is not greatly superior to the tactus eruditis in estimating pressure and that it is infinitely superior for estimating volume, rapidity, regularity, character of pulse wave, etc. In leaky heart, high pressure is a necessary compensation. He deprecates the tendency to use vascular depressants without careful consideration of all the elements of a case.

